# Hypoxia promotes differentiation of adipose-derived stem cells into endothelial cells through demethylation of ephrinB2

**DOI:** 10.1186/s13287-019-1233-x

**Published:** 2019-05-20

**Authors:** Ting Shang, Shuaijun Li, Yun Zhang, Laiya Lu, Lei Cui, Fang Fang Guo

**Affiliations:** 1 Department of Plastic Surgery, Beijing Shijitan Hospital affiliated to Capital Medical University, 10 Tieyi Road, Beijing, China; 2 Department of Plastic and Reconstructive surgery, Zhongda Hospital, Southeast University, 87 Dingjiaqiao street, Nanjing, Jiangsu Province China; 3 Department of Orthopedics, Shanghai Tongji Hospital, Tongji University School of Medicine, 389 Xincun Road, Shanghai, China

**Keywords:** Hypoxia, Adipose-derived stem cells, Vascular endothelial cell, Demethylation, ephrinB2

## Abstract

**Background:**

Delivery of endothelial cells into the ischemic tissue is emerging as an alternative approach in revascularization of injured tissues by means of angiogenesis to restore organ function. Adipose-derived stem cells (ASCs) are a readily accessible source of the mesenchymal stem cell with rapid expansion and multidifferentiation potential. The view has emerged that endothelial cells (ECs) differentiated from ASCs is a step forward for adult vascular repair in regenerative medicine and construction of the blood vessel by tissue engineering approach.

**Methods:**

In this study, differentiation of human ASCs (hASCs) into vascular EC lineage was induced by combined treatment of vascular endothelial growth factor (VEGF) and bone morphogenetic protein-4 (BMP4) under hypoxia condition. The expression of CD31, VEGF-R2, and VE-cadherin was determined by immunofluorescent staining, real-time PCR, and western blot analysis. These differentiated cells acquired functional characteristics of mature ECs as determined by their tube formation ability, DiI-ac-LDL uptake, and nitric oxide secretion in vitro. The methylation status in the proximal promoter CpGs was determined by bisulfite sequencing.

**Results:**

hASCs expressed endothelial cell markers including CD31, VEGF-R2, and VE-cadherin by combined treatment of VEGF and BMP4 under hypoxia condition. These differentiated cells exhibited the angiogenesis potential in vitro, and injection of these differentiated cells enhanced angiogenesis in the ischemic hindlimb of diabetic mice. Furthermore, it was found that hypoxia increased significantly EphrinB2 expression EC differentiation, which is greatly downregulated with EphrinB2 blockage. The methylation status in the proximal promoter CpG results showed that methylation of EphrinB2 promoter decreased in hASCs with exposure to hypoxia.

**Conclusion:**

Our data demonstrate that hASCs can be efficiently induced to differentiate into vascular EC lineages which are mediated by demethylation of ephrinB2 under hypoxia condition.

## Introduction

As a single cell forming the lining layer of blood vessels, vascular endothelial cells (ECs) play an essential role in maintaining vascular homeostasis by responding to an angiogenetic stimulus, regulating vascular permeability, triggering the process of thrombosis, and interacting with multiple components in blood flow [1]. Delivery of endothelial cells into ischemic tissue is emerging as an alternative approach in revascularization of injured tissues by means of angiogenesis to restore organ function. In this context, a potential source of ECs for augmenting vessel growth is adipose-derived stem cells (ASCs), which are readily harvested, relative abundance, and multipotent that are capable of differentiating into three germ layers [2]. Several studies have demonstrated that administration of ASCs into the ischemic tissue improved revascularization with direct participation in vascular structures [3, 4]. In addition, our previous study showed that ASCs can function as a paracrine source of growth factors to augment angiogenesis in ischemic random-pattern skin flaps in a diabetic mouse model [5]. Thus, the view has emerged that ECs differentiated from ASCs are a step forward for adult vascular repair in regenerative medicine and construction of the blood vessel by tissue engineering approach.

Within the process of angiogenesis, a variety of growth factors working as a network regulates differentiation, sprouting, and tube formation of ECs, among which vascular endothelial growth factor (VEGF) acts as a major regulator during the formation of primitive vascular [6]. In addition, bone morphogenetic protein-4 (BMP4) pathway was reported to act synergistically with VEGF in vasculogenesis during embryonic development. According to Boyd et al., the formation of the primitive vascular network in human embryonic stem cell-derived embryoid was remarkably promoted by stimulation with BMP4 [7]. Given that ASCs reside in a native physiological niche with low oxygen, and a lower oxygen tension facilitates MSC differentiation towards EC lineage, we hypothesized that a hypoxia environment can accelerate the acquirement of EC phenotype by ASCs under stimulation with pro-angiogenic factors.

The Eph receptor tyrosine kinase family is the largest family of tyrosine kinases and includes at least 14 Eph receptors and 8 ligands. The Eph/ephrin family is differentially expressed in various human tissues and involved in embryonic vasculature development [8]. EphrinB2, which has been represented as a marker of arterial endothelial cells, was found to be upregulated during physiological and pathological angiogenesis in the adult [9]. Recently, it was reported by Wang et al. that ephrinB2 acts as a key regulator in VEGF-triggered angiogenesis by stimulating internalization of VEGFR3 in primitive EC specification from their progenitors [10]. However, whether ephrin-B2 involves in EC differentiation of ASCs remains to be elucidated.

In the present study, we investigated the effect of combined stimulation of VEGF and BMP4 on differentiation of ASCs towards EC lineage under hypoxia environment. We found that ephrinB2 plays a critical role in regulating EC differentiation by ASCs and that initiation of EphrinB2 expression triggered by low oxygen tension was a result of demethylation of EphrinB2 promoter.

## Materials and methods

### Isolation, culture, characterization, and differentiation of ASCs

Subcutaneous adipose tissue sites were obtained from 5 female donors (at an average age of 28 years) undergoing tumescent liposuction in accordance with procedures approved by the Ethics Committee. The adipose tissue was washed three times with phosphate-buffered saline (PBS) and digested with 0.1% collagenase type I (Gibco, Carlsbad, CA, USA) at 37 °C for 60 min. The digested lipoaspirates were centrifuged at 1200 g for 10 min to obtain a high-density stromal vascular fraction (SVF). The SVF collection was then treated with red blood cell lysing buffer (0.3 g/L ammonium chloride in 0.01 M Tris HCl buffer, pH 7.5, Sigma) for 5 min, centrifuged at 600 g for 10 min, and then filtered through a 40-μm strainer (BD Biosciences, Bedford, MA, USA). The cells were resuspended in Dulbecco’s modified Eagle’s medium (DMEM) supplemented with 10% fetal bovine serum (FBS) and 1% penicillin/streptomycin, and plated in 100-mm culture dishes (Falcon, Oxnard, CA, USA) at a density of (4 × 10^4^/cm^2^ cells), with the medium changed every 2–3 days. When ~ 80~90% confluence was reached, cells were passaged and those at passage 3 were used in the following study. Multilineage differentiation capacity of hASCs was determined by their osteogenic, adipogenic, and chondrogenic differentiation which were detected with Alizarin red, Oil Red O staining, and immunohistochemical staining for collagen type II, respectively.

### Induction of endothelial differentiation

At first, CD31- and flk-1-positive cells were removed by flow cytometry at passage 2. When reaching 70–80% confluence, ASCs at passage 5 were subjected to serum-free starvation for 24 h, followed by cultivation in different media under normoxia (21% O_2_) and hypoxia (2% O_2_) circumstances that assigned into 5 groups as follows: (1) DMEM supplemented with 10% FBS under normoxia as normal control; (2) EGM-2 supplemented with 50 ng/ml rhVEGF, 100 ng/mlBMP4, and 2%FBS under hypoxia; (3) EGM-2 supplemented with 50 ng/ml rhVEGF and 2%FBS under hypoxia; (4) EGM-2 supplemented with 100 ng/ml BMP4 and 2%FBS under hypoxia; (5) EGM-2 supplemented with 50 ng/ml rhVEGF, 100 ng/mlBMP4, and 2%FBS under normoxia; Human umbilical venous endothelia cells (hUVECs) maintained in EGM supplemented with 2%FBS served as a positive control. The culture media was changed every 2 days.

### Immunofluorescence

Cells were fixed in 4% paraformaldehyde for 20 min at room temperature, permeabilized with methanol for 5 min, and blocked in 5% BSA. After PBS rinses, cells were incubated with primary antibodies against CD31, VEGFR2, VE-Cadherin, and EphrinB2 (Santa Cruz Biotechnology) overnight at 4 °C. After washing, cells were incubated with FITC-conjugated secondary antibodies and were viewed by a fluorescence microscope (Nikon, Tokyo, Japan).

For immunofluorescent staining of CD31-positive cells in the ischemic tissue, frozen sections were air-dried and fixed for 45 min in cold 4% PFA, followed by the same permeablization and blocking procedures as cell culture. Tissue sections were incubated with FITC-conjugated anti-CD31 antibody at 4 °C overnight, followed by washes with PBS at room temperature, and counterstained with DAPI (Life Technologies).

### Flow cytometry analysis

For flow cytometry analysis, cells were harvested, fixed for 30 min in ice-cold 2% paraformaldehyde, and washed in flow cytometry buffer (FCB: 1 PBS, 2% FBS, 0.2% Tween 20). Single-cell suspensions of 10^6^ cells/mL were incubated with anti-CD31-FITC, VEGFR2-FITC, and VE-cadherin-PE antibodies for 60 min on ice. After three further washes with PBS, flow cytometry was performed on a FACS Caliber flow cytometer (Becton Dickson, San Jose, CA, USA). In each experiment, an isotype-matched IgG control was also used.

### Western blot analysis

Cells after induction for 14 days were suspended in cell lysis buffer (Fermentas, Vilnius, Lithuania) and sonicated. After centrifugation, the protein content in the supernatants was determined by a BCA protein quantification kit (Pierce Biotechnology). Sixty milligrams of proteins were added to Laemmli sample buffer and boiled for 10 min. Proteins were separated by sodium dodecyl sulfate-polyacrylamide gel electrophoresis (SDS-PAGE) and transferred to polyvinylidene difluoride membranes. The membranes were blocked with 5% dried fat-free milk in Tris-buffered saline containing 0.1% Tween 20. Incubation with primary antibodies was performed at 4 °C overnight. Immunoreactive bands were visualized by using an IRDye 700DX-and IRDye 800CW-conjugated secondary antibody (Rockland Immunochemical, Gilbertsville, PA, USA), and proteins were visualized by the Odyssey system (LI-COR Biosciences, Lincoln, NE, USA). The western blotting results were quantified using Gel-Pro Analyzer (Version 4.5) software.

### Quantitative real-time PCR

Total RNA was extracted by using the RNeasy Mini Kit (Qiagen, Valencia, CA, USA) according to the manufacturer’s protocol. The RNA concentration was determined by optical density at 260 nm (OD260), using a spectrophotometer (Nano drop ND-1000, Wilmington, DE, USA). Complementary DNA (cDNA) was synthesized from RNA using High-Capacity cDNA Reverse Transcription Kits (Applied Biosystems, Foster City, CA, USA). The sequences of the gene-specific primers are shown in Table 1. Briefly, quantitative RT-PCR was performed using Fast Start Universal SYBR Green master (Roche) and CFX Connect Real-Time PCR Detection System (Bio-Rad, Hercules, CA, USA). The expression level of CD31, VEGFR2, VE-Cadherin, and EphrinB2 was analyzed and normalized to β-actin for each cDNA sample.

**Table 1:** Primers for qPCR

Gene	Primers (F=forward; R=reverse)	size (bp)
CD31	F: 5’-AACAGTGTTGACATGAAGAGCC-3’	148
	R: 5’-TGTAAAACAGCACGTCATCCTT-3’	
Flk-1	F: 5’-GGCCCAATAATCAGAGTGGCA-3’	109
	R: 5’-CCAGTGTCATTTCCGATCACTTT-3’	
VE-Cadherin	F: 5’-GATCAAGTCAAGCGTGAGTCG-3’	114
	R: 5’-AGCCTCTCAATGGCGAACAC-3’	
β-actin	F: 5’-ATCATGTTTGAGACCTTCAA-3’	318
	R: 5’-CATCTCTTGCTCGAAGTCCA-3’	

### AC low-density lipoprotein (LDL) uptake

Cells were seeded into 6-well plates at a density of 5 × 10^4^ cells per well and incubated with acetylated low-density lipoprotein DiI complex (10 μg/ml, DiI AcL DL, Invitrogen Corporation, Carlsbad, CA, USA) for 24 h. Following removal of the medium, cells were washed three times with PBS and observed under fluorescent microscopy.

### Capillary-like tube formation assay

After induction for 14 days, 5 × 10^4^ cells in 500 μl of EGM2 were plated onto 4-well Culture Slide (BD Biosciences, San Jose, CA, USA) that has been pre-coated with 150 μl of growth factor-reduced Matrigel (BD Biosciences) per well. Sixteen hours later, the development of capillary-like networks was examined by phase-contrast microscopy. Tube formation was defined as four times in length than its width. Numbers of tubular branches were counted in 10 random fields per well (Image-Pro Plus) as previously reported [3].

### Production of nitric oxide

NO concentration in culture supernatants was assayed by Greiss method according to manufacturer’s protocol. A standard curve was prepared using a 0.1-mM sodium nitrite. The supernatant was collected, mixed with Greiss solution containing sodium nitrite (at concentrations 6.25, 12.5, 25, 50, 100, and 200 μM) and subjected to ELISA analyzer in 540 nm and 630 nm filters.

### Administration of induced hASCs into the ischemic hindlimb in diabetic mice

Female BABL/C-nu/nu mice (4–6 weeks old, 15–25 g) were injected intraperitoneally with 65 mg/kg of streptozotocin (STZ, Sigma, St. Louis, MO, USA) in 0.9% sterile saline daily for 3 days. Mice with mean fasting blood glucose greater than 16.7 mmol/l were used for the creation of unilateral hindlimb ischemia. Briefly, after the femoral artery in the left hindlimb was exposed, a ligation was performed with 3–0 silk suture at the proximal portion of the artery. The distal portion of the saphenous artery, other arterial branches and veins were isolated free and ligated. After surgery, mice were carefully monitored until they have completely recovered from anesthesia. Blood flow in the hindlimb was measured using a Laser Doppler perfusion imager (LDPI; Moor Instrument, Delaware, DE, USA). The ischemic mice were randomly assigned into 3 groups (*n* = 6 in each group) and received intramuscular injection of 4 × 10^6^ of ASCs labeled with fluorescent DiI (V-22885, Molecular Probes, Eugene, OR, USA) in 100 μl saline according to the manufacturer’s recommendations: group 1, ASCs cultured under hypoxia without induction for 14 days; group 2, ASCs induced with VEGF and BMP4 under normoxia for 14 days; and group 3, ASCs induced with VEGF and BMP4 under hypoxia for 14 days. The normal hindlimb without intervention was served as blank control.

### Full-field laser perfusion imager (FLPI)

Detection of blood flow perfusion in the hindlimb was performed at 14 days after cell transplantation using FLPI (Moor instrument, UK) measurements. Briefly, the CCD camera was positioned 31 cm above the limb surface; a display rate of 25 Hz, time constant of 1.0 s, and camera exposure time of 20 ms were set for low-resolution/high-speed images. The contrast images were processed to produce a color-coded live flux image (red denoted high perfusion, blue signified low perfusion). Every measurement lasted at least 1 min, produced 10 image frames, and was acquired after blood flow had been stabilized under anesthesia. Mean values of perfusion were calculated from the stored digital color-coded images. The level of blood flow of the ischemic (left) limb was normalized to that of the non-ischemic (right) limb to avoid data variations caused by ambient light and temperature.

### Bisulfite sequencing analysis

Genomic DNA was isolated using a DNeasy kit (Qiagen, Valencia, CA, USA). Bisulfite genomic sequencing was performed on 1 μg of genomic DNA using an EZ DNA Methylation-Direct Kit (Zymo Research, Irvine, CA, USA) in accordance with the instruction manual. Briefly, bisulfite-modified DNA was amplified using primers designed according to the online MethPrimer software (www.urogene.org/methprimer/). Purified PCR products were subcloned into the pMD19-T vector (TaKaRa, China). Three independent amplification experiments were performed for each sample. Three to four clones from each independent set of amplification and cloning were sequenced, in which a minimum of nine clones were selected for DNA sequencing (BGI, China). Bisulfite sequencing data and C-T conversion rates were analyzed by BIQ analyzer software.

### Statistical analysis

Data were represented as the mean ± standard error of the mean. Quantitative data are presented as the means’ standard deviation (SD). The data were analyzed by two-tailed Student’s *t* test for means’ analysis to compare two data groups or ANOVA to compare three or more data groups. Statistically significant values were defined as *p* < 0.05.

## Results

### Differentiation of hASCs into ECs with the induction of VEGF and BMP4 under hypoxia

**Fig. 1:**
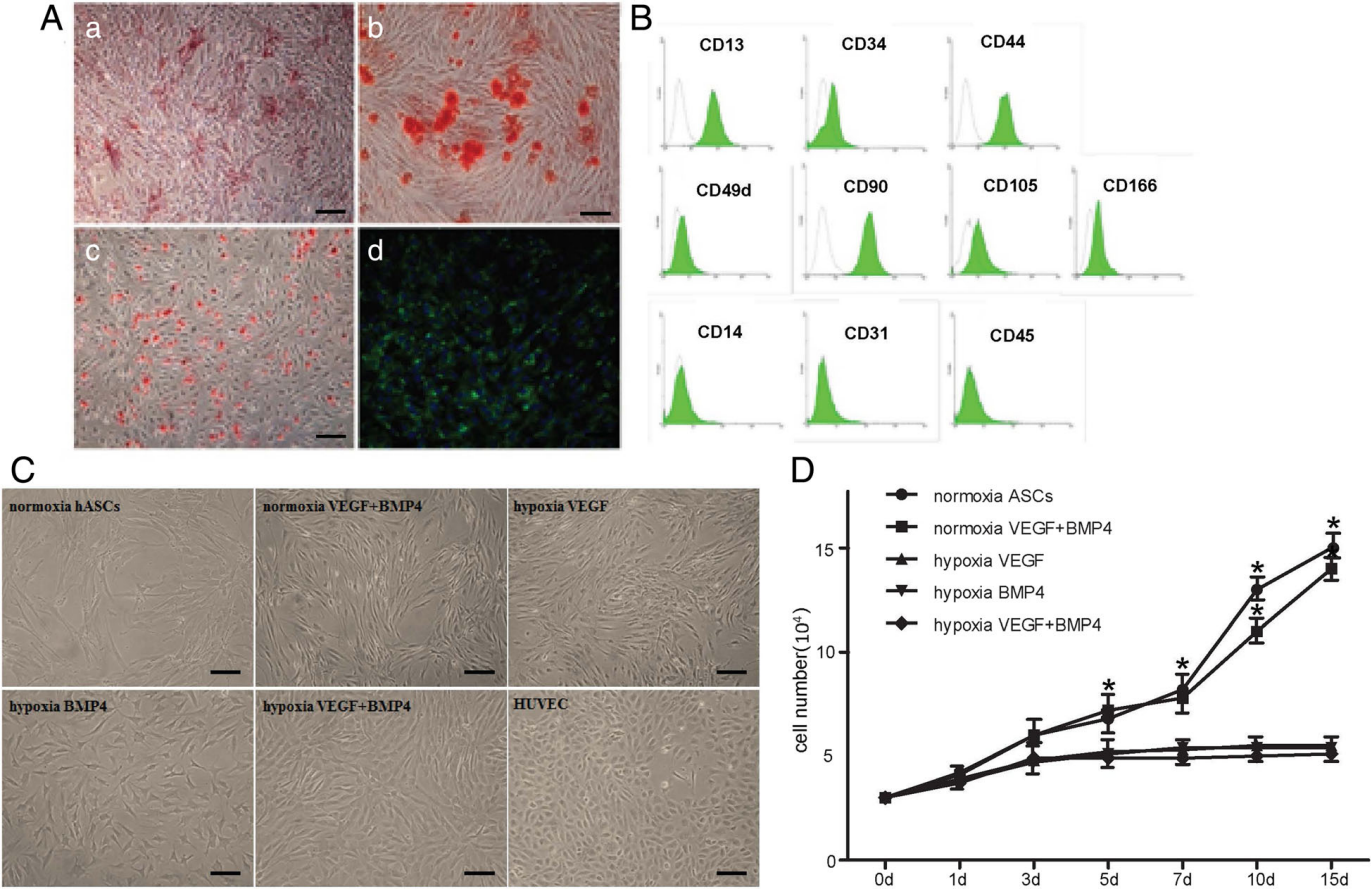
hASCs were induced with a combination of VEGF (50 ng/ml) and BMP4 (100 ng/ml) under either normoxia or hypoxia circumstances for 14 days. **A** Flow cytometry analysis of CD markers in hASCs. **B** Osteogenic, adipogenic, and chondrogenic differentiation of hASCs was determined by ALP and Alizarin red staining, oil red staining, and immunofluorescent staining for collagen type II, respectively. **C** Morphology changes of hASCs under phase-contrast microscope observation. Scale bars: 50 um. Human umbilical vein endothelial cells (HUVECs) served as a positive control. **D** Proliferation of ASCs under different conditions determined by DNA assay using Hoechst 33258 dye

FAC analysis showed that hASCs were positive for CD13, CD44, CD34, CD90, CD105, CD166, and CD49d, but negative for CD14, CD31, and CD45 (Fig. 1A). Osteogenic, adipogenic, and chondrogenic differentiation of hASCs was determined by matrix mineralization, intracellular accumulation of lipid droplet, and collagen type II expression, respectively (Fig. 1B). To investigate whether hASCs could differentiate along endothelial cell pathway, we first treated hASCs with VEGF (50 ng/ml), BMP4 (100 ng/ml) alone, or in a combination of them, respectively, for 14 days under normoxia circumstances. Cells treated with VEGF and BMP4 in combination exhibited a spindle morphology under normoxia (Fig. 1C). It has been reported that hypoxia play a critical role in angiogenesis; we thus transfer hASCs of each group to a hypoxia (2%) environment. Under hypoxia condition for 14 days, hASCs treated with VEGF alone did not show an evident change in their morphology, while BMP4 alone treatment leads to a slight change to polygon shape (Fig. 1C). Subjecting to hypoxia resulted in remarked cell shape change, from spindle to typical cobblestone-like ones in the group that cells were treated with VEGF and BMP4 in combination (Fig. 1C). The proliferation of hASCs subjected to different circumstances within a duration of 14 days was determined by DNA assay using Hoechst 33258 dye. Our results showed that ASCs growing under normoxia, either treated with VEGF, BMP4 alone, or in combination, exhibited a comparable proliferation pattern as cells growing under normoxia alone. Hypoxia significantly suppressed the proliferation of ASCs that treated with VEGF, BMP4 alone, or in a combination of them (Fig. 1D).

**Fig. 2:**
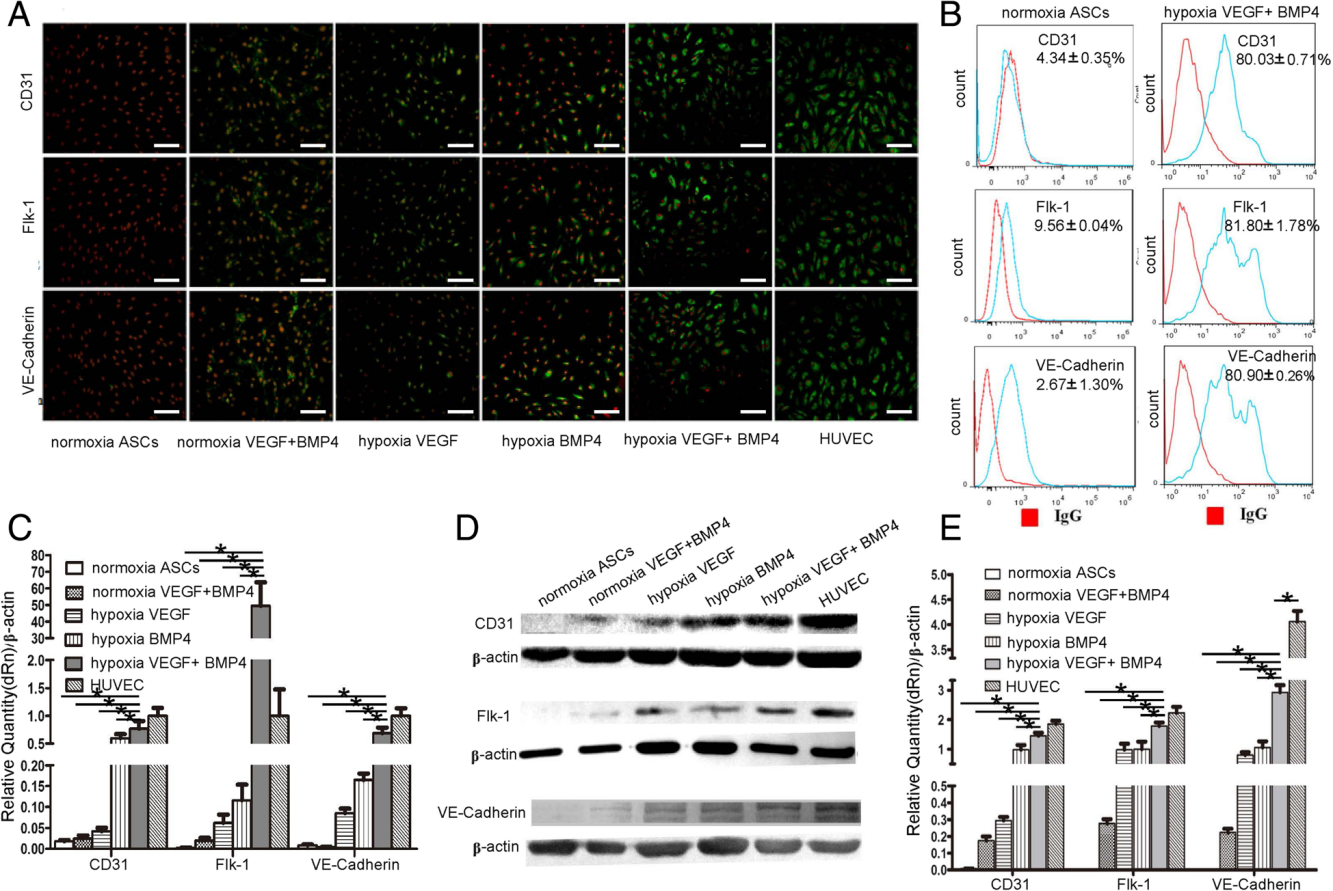
With exposure to either normoxia or hypoxia, expression of CD31, Flk-1, and VE-cadherin in hASCs treated with VEGF (50 ng/ml) and BMP4 (100 ng/ml) for 14 days was determined by **A** immunofluorescent staining, **B** FACS analysis, **C** real-time PCR, and **D** western blot analysis, which was quantified and normalized to the expression levels of β-actin (**E**). Data are presented as mean ± SD. *P < 0.05

To evaluate whether shape change of hASCs subjected to hypoxia is correlated to endothelial cell differentiation, expression of CD31, flk-1, and VE-Cadherin was determined by immunofluorescent staining, which showed that merely under hypoxia circumstances, cells treated with combined VEGF (50 ng/ml) and BMP4 (100 ng/ml) showed an evident increase compared to their corresponding controls (Fig. 2A). Furthermore, FACs analysis indicated that, as a result of exposure to hypoxia, CD31-, flk-1-, and VE-cadherin-positive cells reached 80.03 ± 0.71%, 81.80 ± 1.78% and 80.90 ± 0.26%, respectively, in hASCs treated with the combination of VEGF and BMP4 (Fig. 2A). The expression of these markers was further assessed by real-time PCR and western blot, which revealed either mRNA levels or protein levels of these EC markers were greatly upregulated under hypoxia (Fig. 2C, D).

**Fig. 3:**
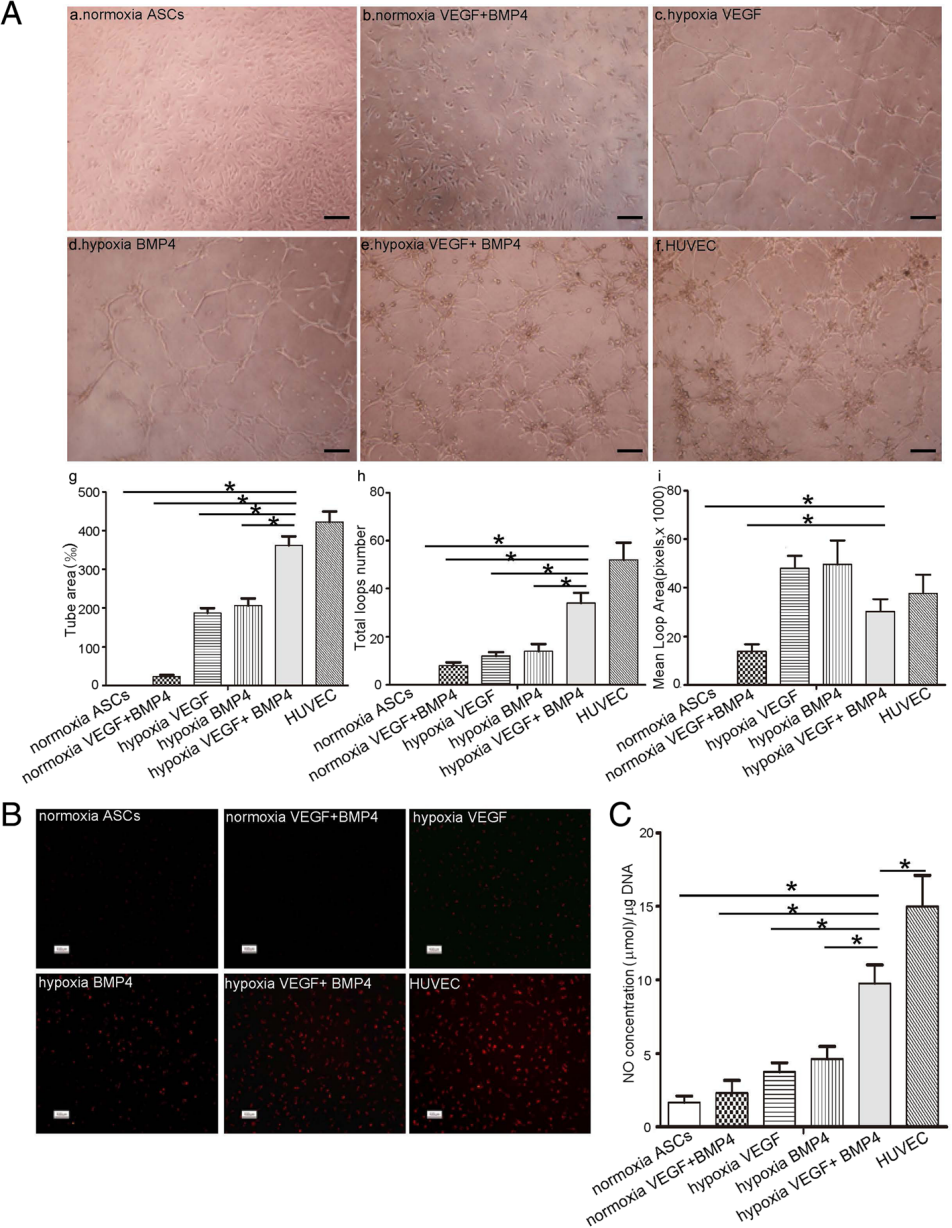
Functional characteristic of vascular ECs differentiated from hASCs after treatment with VEGF (50 ng/ml) and BMP4 (100 ng/ml) for 14 days under hypoxia. **A** Tube formation (a–f) of induced hASCs on Matrigel and quantitative calculation of tube area (g), total loops number (h), mean loop area (i). Scale bar: 50 um. **B** Uptake of Dil-Ac-LDL by induced hASCs under indicated conditions. Scale bar: 20 um. **C** Nitric oxide production of induced hASCs. HUVECs served as a positive control. Data are presented as mean ± SD. *P < 0.05

Next, we examined the angiogenic ability of hASCs in an in vitro model of tube sprouting. Cells from different groups were seeded within three dimensions for 16 h, and the formation of the tubular structure and spontaneous branching of neo-generated tubes were calculated. EC-hASCs showed robust capillary tube formation and branching in 3D gels. As determined by tube area and total loop number calculation, hypoxia-exposed hASCs treated with VEGF and BMP4 exhibited the most angiogenesis potential among different groups (Fig. 3A).

To ascertain whether EC-hASCs are able to incorporate ac-LDL, a function of which has been used to characterize endothelial cells, we incubated cells with Dil-ac-LDL and subsequently observed under fluorescence microscopy. As shown in Fig. 3B, take up of ac-LDL was remarked increased in EC-hASCs as compared with cells in other groups. Furthermore, EC-hASCs exhibited a comparable capacity in NO secretion as that of HUVEC controls, which is significantly higher than that of other groups (Fig. 3C).

### Injection of induced hASCs enhanced angiogenesis in the ischemic hindlimb of diabetic mice

**Fig. 4:**
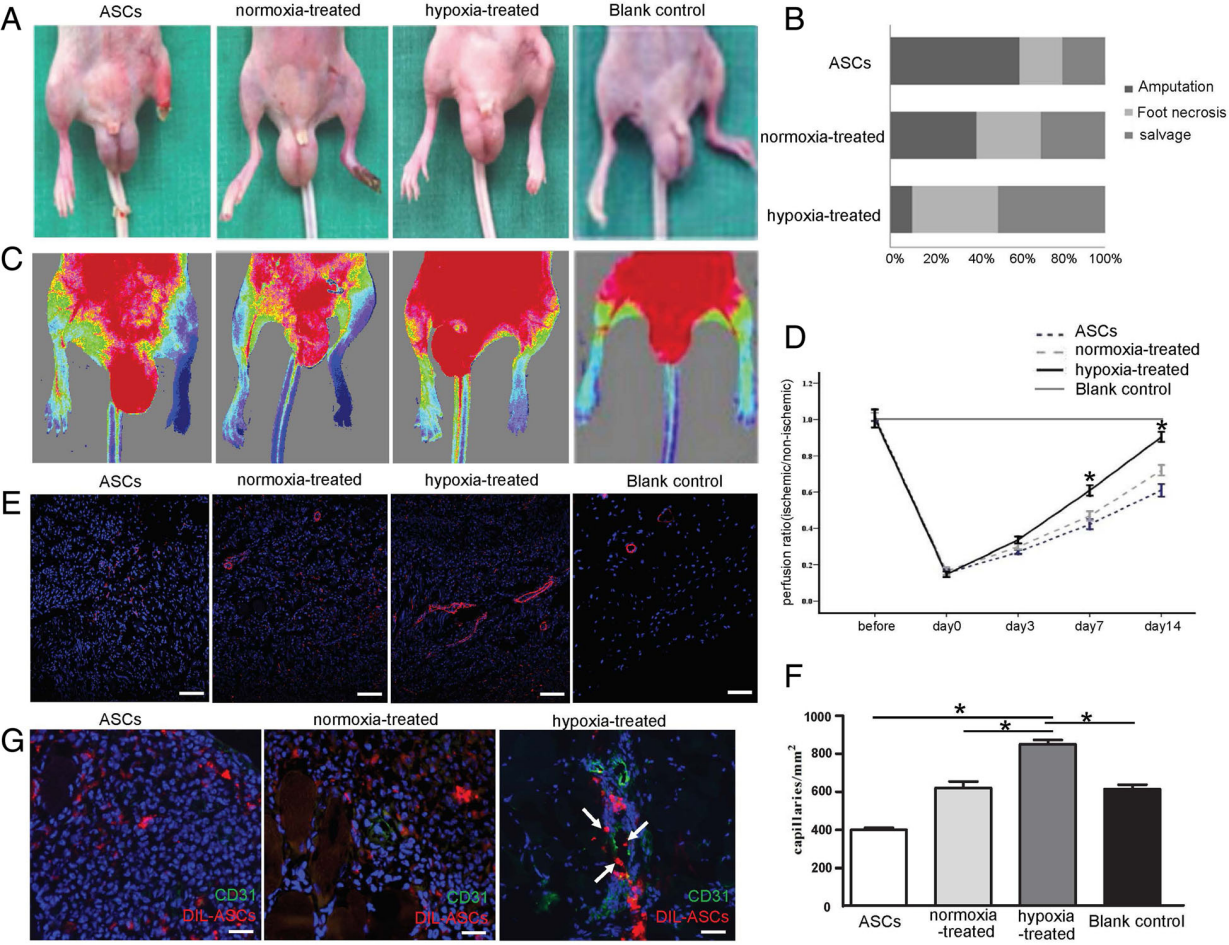
Detection of blood flow perfusion in the hindlimb of diabetic mice subjected to femoral artery ligation as well as hASC transplantation after 14 days. **A** Representative gross view of the ischemic hindlimb received transplantation of hASCs. **B** Quantitative analysis of amputation, foot necrosis, and limb salvage of mice in each group (*n* = 6). Blank control: normal hindlimb without intervention. **C** Representative images of blood flow perfusion detected by full-field laser perfusion imager. **D** Quantification of blood flow recovery presented as the ratio of ischemic to non-ischemic limb (from the knee to toe) in blood blow. **E** Immunofluorescent staining of CD31-positive cells in the ischemic subcutaneous tissue. Scar bar = 20 um. **F** Quantification of percentage of CD31-positive cells identified by immunofluorescent staining. Data are presented as mean ± SD. *P < 0.05. **G** Co-localization of CM-DIL-labeled hASCs and endothelial cells immunostained with anti-CD31 antibody. Arrowheads indicate co-localization of label hASCs with CD31-positive endothelial cells. Scar bar = 20 um

As we demonstrated that endothelial cells differentiated from hASCs possessed comparable angiogenesis capacity in the in vitro model, we thus explored their regenerative potential using a limb ischemia model. Hindlimb ischemic injuries were induced in recipient diabetic mice by ligation of the femoral artery, hASCs induced with VEGF and BMP4 together that either exposed to hypoxia or normoxia for 14 days were injected intramuscularly into the ischemic limbs. On day 14 after ligation, the occurrence of amputation, foot necrosis, and salvage in each group was calculated, and blood flow of the hindlimb was evaluated with FLPI. Injection of EC differentiated from hASCs exposed to hypoxia significantly improved limb salvage with reduction of amputation and foot necrosis occurrence as compared with mice injected with cells of controls (Fig. 4A). Further, we quantified analysis amputation, foot necrosis, and limb salvage of mice in each group (*n* = 6) (Fig. 4B). Accordingly, FLPI analysis showed significant improvement of blood flow in the limbs with transplantation of EC-hASCs (Fig. 4C). Quantification of blood flow recovery was performed as the ratio of the ischemic to the non-ischemic limb (from the knee to toe) in blood blow (Fig. 4D).

Immunofluorescent staining of CD31 revealed an increased distribution of the capillary vessels in the subcutaneous tissue from EC-hASC-treated group (Fig. 4E). According to the result of immunofluorescent staining, the percentage of CD31-positive cells was identified by quantitative analysis (Fig. 4F). These results suggest that EC-hASCs are effective in promoting angiogenesis in the ischemic tissue in vivo. To determine whether injected ASCs participated in angiogenesis of the ischemic tissue, we labeled cells with fluorescent CM-DiI before transplantation. On day 14 after injection, labeled hASCs that induced under hypoxia were observed to be localized in a pattern of aggregation close to vasculature, some of which was incorporated in the vascular wall, indicating that ECs induced from hASCs directly involved in neo-generation of capillary vessels. However, little labeled cells could be detected in the wall of the blood vessels with the injection of either hASCs or induced hASCs exposed to normoxia (Fig. 4G).

### EphrinB2 mediates endothelial cell differentiation of hASCs

**Fig. 5:**
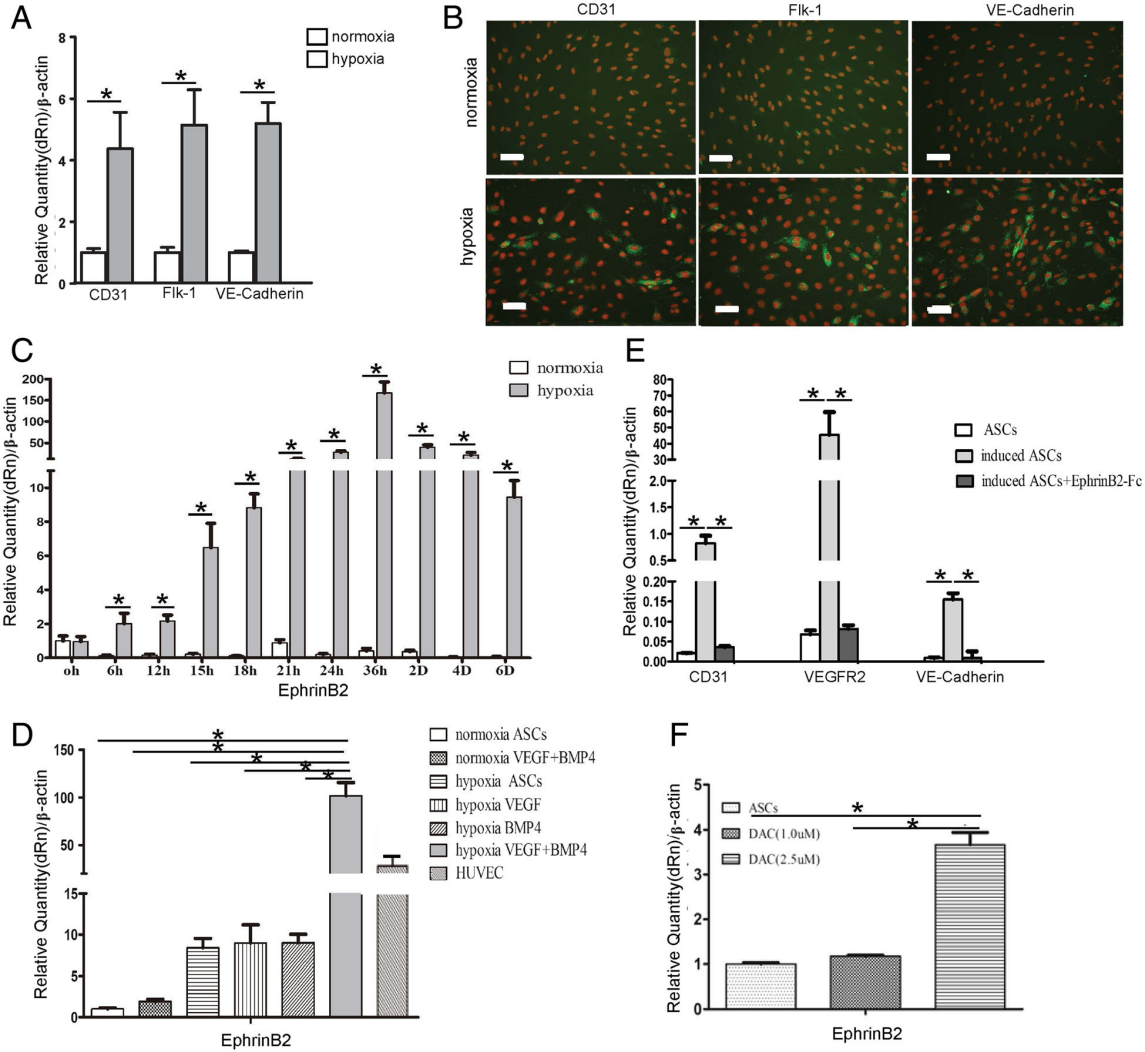
Differentiation of hASCs into vascular ECs is modulated through demethylation of ephrinB2 with exposure to hypoxia. Expression of CD31, Flk-1, and VE-cadherin under hypoxia condition was determined by **a** real-time PCR and **b** immunofluorescent staining. Scale bar: 50 um. **c** Real-time PCR analysis shows that hypoxia alone enhances expression of ephrinB2 in hASCs in a time-dependent manner. **d** Real-time PCR data showing that ephrinB2 expression was significantly increased in hASCs treated with VEGF and BMP4 in combination under hypoxia. **e** Expression of CD31, flk-1, and VE-cadherin was significantly decreased with addition of neutralizing agent against ephreinB2 as determined by real-time PCR. **f** Expression of ephrinB2 was detected by real-time PCR at 72 h after addition of DAC (1.0 uM, 2.5 uM), respectively. Data are presented as mean ± SD. *P < 0.05

Given that hASCs adopted a differentiation pathway towards the endothelial cell merely under hypoxia condition, we next evaluated at what degree did hypoxia affect EC differentiation by hASCs. As determined by real-time PCR and immunofluorescent staining, hypoxia stimulation resulted in an enhanced expression of CD31, Flk-1, VE-cadherin after 14 days, which shows no significance as compared with cells treated under normoxia alone (Fig. 5A, B). It was reported that EphrinB2 is a key regulator of VEGF signal pathway and thereby controls vascular development and postnatal neovascularization in tissue repair. We examined the expression of EphrinB2 under hypoxia condition. We found that hypoxia alone can increase EphrinB2 expression in a time-dependent manner, reaching its highest level after 36 h of exposure to hypoxia. Again, EphrinB2 expression was observed unchanged in normoxia (Fig. 5C). Furthermore, in the process of endothelial cell differentiation by hASCs, real-time PCR results showed that the expression of EphrinB2 was greatly upregulated in hASCs induced by VEGF and BMP4 under hypoxia, reaching a comparable level to that in HUVECs. It is noticeable that exposure to hypoxia resulted in relatively increase of EphrinB2 expression in different groups, whereas it is retained unchanged in cells exposed to normoxia (Fig. 5D). More importantly, with the addition of neutralizing EphrinB2-Fc as the previous report, the expression of CD31, VEGF-R2, and VE-cadherin distinctly decreased to similar levels of hASC controls without induction (Fig. 5E). To further investigate, the role of DNA methylation on EphrinB2 transcription that has been proved to mediate EC differentiation by hASCs, we examined the expression of EphrinB2 in EC-hASCs with addition of DAC (5-aza-dC), an inhibitor of DNA methyltransferase. Treatment with DAC at a concentration of 2.5 μM for 72 h clearly enhanced the expression of EphrinB2 in hASCs (Fig. 5F).

**Fig. 6:**
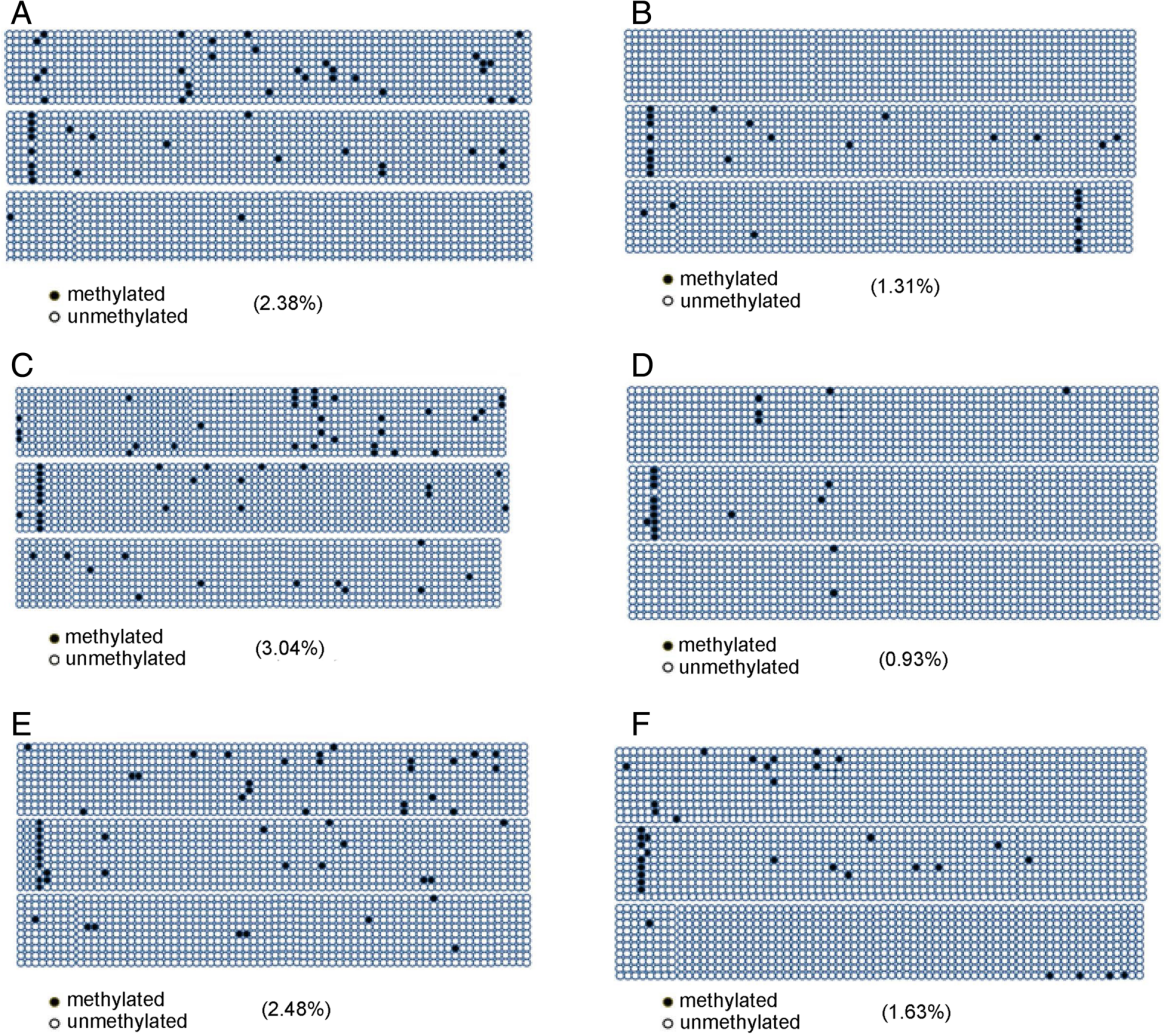
hASCs that harvested from 3 individuals were exposure to hypoxia (**b**, **d**, **f**) and normoxia (**a**, **c**, **e**) for 36 h, respectively. The methylation level of CpG sites in ephrinB2 promoter was calculated with EMBOSS Cpgplots. Each black and open cycle represents a methylated and unmethylated CpG dinucleotide, respectively

Bisulfate sequencing analysis was performed to investigate the methylation profile in the promoter regions of EphrinB2. The putative CpG islands were calculated with the EMBOSS CpG plot program. The DNA methylation status of the EphrinB2 promoter-associated CpG island (from − 537 to and 863) was examined in three different individual ASCs treated by hypoxia for 36 h. As shown in Fig. 6, bisulfite sequencing analysis shows that EphrinB2 were demethylated greatly after hypoxia exposure (Fig. 6B, D, and F). However, EphrinB2 were retained methylated status after exposed to normoxia (Fig. 6A, C, and E). These results indicate that the EphrinB2 promoter region examined is strongly methylated in ASCs but displays demethylation in differentiation into endothelial cells.

## Discussion

As a readily obtained adult mesenchymal stem cell, adipose-derived stem cells have been characterized in many studies by their intrinsic characteristics of self-renewal, long-term viability, multilineage differentiation capacity into cells of mesodermal origin (osteoblasts, chondrocytes, and adipocytes), and even non-mesodermal lineages (such as hepatocytes [11]). The number of ASCs in the adipose tissue is reported 100–500 times larger than that of bone marrow resident mesenchymal stem cell [12], which makes ASCs a promising cell source in therapeutic revascularization and regenerative medicine. In this study, we have generated vascular ECs from human ASCs. The induced cells exhibited EC surface marker profile and acquired mature EC functions of LDL uptake and tubular formation in vitro. More importantly, ECs that differentiated from hASCs improved substantially the revascularization when injected topically in an ischemic limb model.

The role of VEGF in modulating EC fate determination during the process of angiogenesis in ischemic or injured tissue administrated with stem cells is well documented. However, treatment with VEGF alone shows little effect on differentiation of ASCs towards endothelial cells. Given that the role of BMP4 in vascular and hematopoietic development was recognized recently, we chose BMP4 to act synergistically with VEGF to induce ASCs to differentiate to vascular ECs. ASCs failed to adopt an EC phenotype even under the combined stimulation of BMP4 and VEGF in our experiment. In fact, it has been previously revealed that oxygen concentration in the physiology niche that ASCs reside in the fat tissue is as low as 3% [13], indicating that a hypoxia microenvironment could be beneficial for differentiation of ASCs into vascular ECs. Furthermore, the expression of angiogenic growth factors such as VEGF and FGF-2 in ASCs were significantly increased under hypoxia conditions. We thus induced ASCs with a combination of VEGF and BMP4 under hypoxia environment. Exposure to hypoxia resulted in a significant increase in the expression of EC markers including CD31, flk-1, and VE-cadherin. However, either VEGF or BMP4 alone combined with hypoxia exposure shows no inductive effect on ASC differentiation. The induced ASCs under hypoxia not only exhibited the EC markers but also acquired EC functions including LDL uptake, NO production, and tubular formation in 3D culture in vitro. However, hypoxia itself did not stimulate the expression of EC markers in hASCs. These findings are consistent with the findings of other authors assessing the differentiation capacity of MSCs to endothelial cells under low oxygen tension [14, 15].

A number of studies have shown that ASCs secrete significant quantities of angiogenic factors. These findings further encouraged a series of in vivo studies that focused on evaluating the therapeutic potential of cells based particularly on their paracrine and angiogenic effects [16]. In this study, a significant increase of revascularization was found in an ischemic hindlimb model that received a topical injection of induced ASCs. Compared with animals received administration of non-induced ASC controls and ASCs induced with VEGF and BMP4 in combination under normoxia, mice administrated with hASCs induced by VEGF and BMP4 in combination under hypoxia showed the highest salvage and lowest amputation rate. Evidence was also provided for the restored blood flow as observed by laser perfusion analysis. Furthermore, by labeling cells with DIL, we found the incorporation of labeled EC-hASCs in the vascular wall after injection for 14 days, indicating a direct participation of EC-hASCs in angiogenesis within the ischemic tissue. Together with the histological evidence showing elevated capillary vessel regeneration, these data clearly show that induced hASCs with exposure to hypoxia are capable of stimulating neo-vessel formation in ischemic tissues.

During both developmental and pathological angiogenesis, EphrinB2 has been revealed to play crucial roles in regulating VEGF receptor-2 internalization and downstream signaling to direct filopodial extension [17]. By generating genetic mice in which ephrinB2 is specifically inactivated in the endothelium, Gerety et al. [18] found that such a vascular-specific deletion of ephrinB2 impairs early embryonic angiogenesis and cause lethality. In a recent study, it is discovered that EphrinB2 controls vessel pruning through STAT1-JNK3 signaling which is VEGF/VEGFR2-independent [19]. Binding of epherinB2 to its corresponding EphB receptors trigger an EphB-mediated forward as well as ephrinB2-stimulated reverse signaling during cell-to-cell contact in border formation between arterial and venous endothelial cells [20]. However, to our knowledge, no report has been found whether ephrinB2 take part in the regulation of vascular endothelial differentiation by adult MSCs. In this study, we detected a significant increase in the expression of ephrinB2 in hASCs treated with VEGF and BMP4 in combination under hypoxia. It is interesting to notice that exposure to hypoxia individually enhanced ephrinB2 expression. Adding either VEGF or BMP4 alone did not further enhance the expression of ephrinB2, and indicating low oxygen concentration may act as a prerequisite in differentiation into EC lineage of hASCs. In fact, upregulation of ephrins of both A and B subclasses and their Eph receptors have been observed in hypoxic skin flap [21]. Moreover, Sohl et al. found that the expression of ephrinB2 is significantly increased with hypoxia exposure via a hypoxia-inducible factor-independent mechanism, which is mediated through the binding of Sp1 to the ephrinB2 promoter [22]. In this study, with the addition of neutralizing EphrinB2-Fc, the expression of CD31, VEGF-R2, and VE-cadherin was distinctly decreased to similar levels of hASC controls under normoxia condition. Taken together, it seems that differentiation of hASCs into EC phenotype was fulfilled synergistically with angiogenic factors under hypoxia environment, which is mediated by ephrinB2 activation. In accordance with our findings, a previous study demonstrated that ephrinB2 expression can be induced by BMP through an Alk1-BMPRII/ActRII-ID1/ID3-dependent pathway [23].

During cell fate determination, epigenetic modifications among which DNA methylation in the promoter regions has been shown to play critical roles in regulating the expression of lineage-specific genes. For example, inhibition of DNA methylation promotes greatly the expression of EC marker genes in EC differentiation of embryonic stem cells [24]. It is noticeable that a hypermethylation in the proximal promotor CpGs of VEGF-A, BMP4 was detected before EC differentiation by embryonic stem cells. Demethylation of GATA-2, GATA-3, and eNOS promoters in cultured human ESCs has been shown to promote their differentiation towards mature ECs [25]. However, whether epigenetic modulation is responsible for ephrinB2 expression and thereby trigger the EC differentiation of hASCs remains unclear. In this study, we found that the addition of an inhibitor of DNA methyltransferase enhanced greatly the expression of ephrinB2 in hASCs, indicating that DNA methylation is responsible for ephrinB2 expression in hASCs. We further detected the methylation status of ephrinB2 in hASCs. Using bisulfite sequencing analysis, we found a high methylation within ephrinB2 promoter region in hASCs. With exposure to hypoxia, a significant demethylation was detected in hASCs. The mechanism regulating demethylation of the ephrinB2 promoter by hypoxia exposure in hASCs needs to be elucidated. In accordance with our study, it was recently reported that hMSCs with stable knockdown of Dnmt1/ Dnmt3a were highly angiogenic and expressed several arterial-specific transcription factors and marker genes [26].

## Conclusion

In conclusion, this study demonstrates that, with exposure to hypoxia, a combination of VEGF and BMP4 induces the differentiation of hASCs into vascular endothelial cells which is under control of ephrinB2 activation. Hypoxia acts as a prerequisite for EC differentiation by hASCs and induces demethylation of ephrinB2 promoter regions. Local injection of the induced ECs preconditioned with hypoxia exposure accelerates revascularization in an ischemic limb model. Induction of hASC differentiation into ECs provides a promising alternative of cell source for vascular tissue engineering and other therapeutic revascularization in the future.

## Abbreviations

BMP4: Bone morphogenetic protein-4
DIL: 1,1′-Dioctadecy1-3,3,3′,3′-tetramethylindocarbocyanine perchlorate
EC: Endothelial cell
FBS: Fetal bovine serum
FCB: Flow cytometry buffer
FLPI: Full-field laser perfusion imager
hASCs: Human adipose-derived stem cells
hUVECs: Human umbilical venous endothelia cells
LDL: Low-density lipoprotein
NO: Nitric oxide
PBS: Phosphate-buffered saline
VEGF: Vascular endothelial growth factor
